# Treatment of cerebral glioblastoma-caused bruxism with mirtazapine: a case report

**DOI:** 10.1186/s40981-020-00329-4

**Published:** 2020-03-21

**Authors:** Mohsen Khosravi

**Affiliations:** Department of Psychiatry and Clinical Psychology, Baharan Psychiatric Hospital, Zahedan University of Medical Sciences, Zahedan, IR 9813913777 Iran

**Keywords:** Bruxism, Glioblastoma, Mirtazapine

## Abstract

**Background:**

Bruxism refers to grind or gnash the opposing rows of upper and lower molar teeth. It is important to treat bruxism as a factor that can influence sleep quality, quality of life, and mental status in patients with malignancy.

**Case presentation:**

A 41-year-old male developed bruxism secondary to cerebral glioblastoma. l-dopa, gabapentin, clonazepam, clonidine, baclofen, buspirone, or propranolol were not effective. Mirtazapine, prescribed for side effects of chemotherapy, was effective for bruxism, which was disappeared within 3 weeks.

**Conclusions:**

Mirtazapine was effective for treating bruxism as well as chemotherapy complications.

## Background

Bruxism is a repetitive masticatory muscle activity characterized by gnashing and grinding the teeth, and/or bracing, or thrusting mandible [1]. It has been demonstrated that bruxism has two circadian manifestations: sleep bruxism, and awake or diurnal bruxism [1]. According to fMRI evidence, diurnal tooth clenching is known to be related to activation of the bilateral sensorimotor cortex, supplementary motor area, dorsolateral prefrontal cortex, and the posterior parietal cortex [2]. This result suggests further evidence for a complex central mechanism involved in bruxism behavior [2]. Recent findings have shown that glioblastoma originated from the above-mentioned regions that can be involved in the incidence and pathogenesis of bruxism [3]. Furthermore, There is some evidence that chemotherapy may lead to or exacerbates bruxism [4]. The quality of life in such patients becomes increasingly important due to poor survival of this malignancy (14 months on average) [5, 6]. Therefore, it is important to treat bruxism as a factor that can influence sleep quality, quality of life, and mental status in these patients [7]. Although a range of drugs including muscle relaxants, sedatives anxiolytics, dopaminergic agents, and antidepressants has been suggested for pharmacological treatment [8], there have been no definitive treatments. We report a case of awake bruxism caused by the development of cerebral glioblastoma, which was successfully treated by a noradrenergic and specific serotonergic antidepressant, mirtazapine.

## Case presentation

A 41-year-old male referred to our hospital was complaining of headache, amnesia, and left arm paresthesia. He also stated the onset of awake bruxism. He had been treated at a psychiatric clinic with a diagnosis of post-traumatic stress disorder after a terrorist attack and receiving risperidone, carbamazepine, citalopram, zolpidem, and melatonin. Brain MRI demonstrated a tumor (34 × 37 mm) in the right frontal and parietal lobes accompanied with central necrosis and peripheral edema. A diagnosis of glioblastoma was made and he received radiotherapy and chemotherapy with temozolamide. l-dopa, gabapentin, clonazepam, clonidine, baclofen, buspirone, and propranolol were not effective for bruxism. Mirtazapine 15 mg/day was started and increased to 30 mg/day due to exacerbation of nausea, anorexia, insomnia, and amnesia following chemotherapy. It was effective for reducing the severity of bruxism as well as other symptoms. Bruxism disappeared completely within 3 weeks and did not recur despite the enlarged tumor size after sessions of radiotherapy and chemotherapy (38 × 40 mm).

## Discussion and conclusions

Bruxism is the most frequently occurring oral movement disorder, and psychological factors and pathophysiological factors have been suggested as its etiologies [9]. Because the complications of bruxism, such as tooth wear, masticatory muscle pain, and insomnia significantly impairs the quality of life, prompt treatment is required. However, only a few controlled studies have been conducted for examining the effect of therapeutic agents [10]. Among antidepressants, amitriptyline and selective serotonin reuptake inhibitors (SSRIs) may exacerbate sleep bruxism [8]. On the other hand, there have been no data regarding the efficacy of other antidepressants including mirtazapine on bruxism [8]. In our patient, mirtazapine was effective for suppressing side effects of chemotherapy as well as bruxism. The effect of mirtazapine in the treatment of bruxism is related to unique mode of biochemical CNS action [11]. In fact, Mirtazapine increases dopaminergic neurotransmission in the prefrontal cortex by (1) 5-HT2A and 5-HT2C receptors blockade, (2) 5-HT1A receptor activation, and (3) an elevation in noradrenaline levels, which may contribute to the effect in our patient [12, 13]. This subject further reinforces the dopamine dysregulation hypothesis in the pathogenesis of bruxism [8].

In conclusion, mirtazapine was effective for treating bruxism as well as chemotherapy complications. However, wider investigations are necessary in this field through randomized controlled trials.

## Data Availability

Not applicable
